# Histone H3 N-Terminal Lysine Acetylation Governs Fungal Growth, Conidiation, and Pathogenicity through Regulating Gene Expression in *Fusarium pseudograminearum*

**DOI:** 10.3390/jof10060379

**Published:** 2024-05-25

**Authors:** Hang Jiang, Lifang Yuan, Liguo Ma, Kai Qi, Yueli Zhang, Bo Zhang, Guoping Ma, Junshan Qi

**Affiliations:** 1 Shandong Key Laboratory of Plant Virology, Institute of Plant Protection, Shandong Academy of Agricultural Sciences, Jinan 250100, China; jhfor724@163.com (H.J.); maliguo809@163.com (L.M.); qishi20080318@163.com (K.Q.); yueligaoxing@163.com (Y.Z.); zbo8341@163.com (B.Z.); maguopingapple@163.com (G.M.); 2 Shandong Academy of Grape, Shandong Academy of Agricultural Sciences, Jinan 250100, China; ylifang1225@126.com

**Keywords:** histone acetylation, Fusarium crown rot, phytopathogen, H3 N-terminal lysine residues, gene expression

## Abstract

The acetylation of histone lysine residues regulates multiple life processes, including growth, conidiation, and pathogenicity in filamentous pathogenic fungi. However, the specific function of each lysine residue at the N-terminus of histone H3 in phytopathogenic fungi remains unclear. In this study, we mutated the N-terminal lysine residues of histone H3 in *Fusarium pseudograminearum*, the main causal agent of Fusarium crown rot of wheat in China, which also produces deoxynivalenol (DON) toxins harmful to humans and animals. Our findings reveal that all the FpH3^K9R^, FpH3^K14R^, FpH3^K18R^, and FpH3^K23R^ mutants are vital for vegetative growth and conidiation. Additionally, FpH3K14 regulates the pathogen’s sensitivity to various stresses and fungicides. Despite the slowed growth of the FpH3^K9R^ and FpH3^K23R^ mutants, their pathogenicity towards wheat stems and heads remains unchanged. However, the FpH3^K9R^ mutant produces more DON. Furthermore, the FpH3^K14R^ and FpH3^K18R^ mutants exhibit significantly reduced virulence, with the FpH3^K18R^ mutant producing minimal DON. In the FpH3^K9R^, FpH3^K14R^, FpH3^K18R^, and FpH3^K23R^ mutants, there are 1863, 1400, 1688, and 1806 downregulated genes, respectively, compared to the wild type. These downregulated genes include many that are crucial for growth, conidiation, pathogenicity, and DON production, as well as some essential genes. Gene ontology (GO) enrichment analysis indicates that genes downregulated in the FpH3^K14R^ and FpH3^K18R^ mutants are enriched for ribosome biogenesis, rRNA processing, and rRNA metabolic process. This suggests that the translation machinery is abnormal in the FpH3^K14R^ and FpH3^K18R^ mutants. Overall, our findings suggest that H3 N-terminal lysine residues are involved in regulating the expression of genes with important functions and are critical for fungal development and pathogenicity.

## 1. Introduction

Fusarium crown rot (FCR) is one of the most important wheat diseases, which occurs predominantly in arid and semi-arid regions worldwide [1]. It has resulted in serious production losses in wheat-growing regions of China in recent years [2,3,4]. In some years and regions with severe outbreaks, crown rot disease can cause wheat yield losses of up to 10% to 30%. Planting cereal seed inoculated with conidia can cause yield losses of up to 85% in Australia [5]. *Fusarium pseudograminearum* is the primary causal agent of FCR in Huanghuaihai region [2]. Additionally, *F. pseudograminearum* also causes wheat scab and maize seedling blight [6,7,8], and produces several fungal mycotoxins such as deoxynivalenol (DON) and nivalenol (NIV). These toxins can function as pathogen virulence factors and also have the potential to contaminate food and feed derived from infected plants, posing serious threats to human and animal health [9].

The nucleosome serves as the basic unit of chromatin, comprising four histones (H2A, H2B, H3, and H4) in eukaryotes [10,11]. DNA is wrapped around a histone octamer consisting of two copies each of H2A, H2B, H3, and H4. The N-terminal regions of histones are flexible and undergo various post-translational modifications (PTMs) [12]. In the histone N-terminal region, several conserved lysine (K) residues are targets for histone acetylation, a key histone PTM regulated by histone acetyltransferases (HATs) and deacetylases (HDACs) [13,14]. This modification plays critical roles in maintaining normal cellular functions such as cell cycle regulation, cell proliferation, apoptosis, gene transcription regulation, tumorigenesis, and cancer progression [15,16,17,18].

K9, K14, K18, and K23 are four crucial lysines in the N-terminus of histone H3 [12], and changes in the acetylation levels of H3K9, K14, K18, and K23 lead to severe functional defects. In *Drosophila*, H3K9 regulates chromatin organization and transcription in heterochromatin, as well as genome DNA replication initiation [19], while H3K14 plays an important role in chromatin accessibility and tissue-specific gene regulation [20]. In *Sacchromyces cerevisiae*, H3K9 is involved in temperature and DNA damage sensitivities [21], whereas H3K14 acetylation is important for rDNA silencing and replicative aging [22]. The mutation of H3K9 results in a slower vegetative growth rate in *Neurospora crassa* [23]. In *Fusarium graminearum*, the deletion of *FgGCN5* and *FgSAS3* results in decreased H3 acetylation levels and serious defects in growth, conidiation, sexual reproduction, secondary metabolism, pathogenicity, and DON production [15]. In *Fusarium fujikuroi*, *Zymoseptoria tritici*, and *Aspergillus flavus*, *GCN5* or *SAS3*, which acetylate histone H3, have a strong impact on vegetative growth, conidiation, secondary metabolism biosynthesis, stress responses, virulence, and the expression regulation of effector genes [17,24,25]. Therefore, histone H3 N-terminal lysines play critical roles in gene expression and cell development processes.

HATs generally acetylate multiple lysines of histones and nonhistone proteins in organisms; however, the functions of the individual lysines of histone H3 N-terminus remain unknown in phytopathogenic fungi. In this study, we mutated H3K9, H3K14, H3K18, and H3K23 to arginines (R) in situ, and characterized their function in vegetative growth, conidiation, sensitivity to different stresses and fungicides, and pathogenicity in *F. pseudograminearum*. FpH3^K14R^ and FpH3^K18R^ mutants exhibited serious defects in vegetative growth and plant infection. In infected wheat kernels, the FpH3^K18R^ mutant showed significantly reduced DON production, while the FpH3^K9R^ mutant showed significantly increased DON production. The FpH3^K14R^ mutant was involved in response to sodium dodecyl sulfate (SDS), H_2_O_2_, Congo red, NaCl, fludioxonil, phenamacril, and carbendazim. Through transcriptome sequencing, we identified 1863, 1400, 1688, and 1806 genes, including many that play important roles in growth, conidiation, DON biosynthesis, and pathogenicity, that were downregulated in FpH3^K9R^, FpH3^K14R^, FpH3^K18R^, and FpH3^K23R^ mutants, respectively. Our results indicate that histone H3 N-terminal lysine acetylation is crucial for gene transcription responsible for growth, conidiation, pathogenicity, and DON production in *F. pseudograminearum*.

## 2. Materials and Methods

### 2.1. Histone H3 Identification

The protein sequence of yeast histone H3 (YBR010W or YNL031C) was obtained from the *Saccharomyces* Genome Database (www.yeastgenome.org) and used to search against the genome database of *F. pseudograminearum* strain CS3096 (GCA_000303195.1) at EnsemblFungi (fungi.ensembl.org/index.html, accessed on 23 February 2021) by BlastP. Subsequently, the H3 protein sequence of *F. pseudograminearum* was used to search against the *Saccharomyces* genome database for verification.

### 2.2. Strains and Culture Conditions

The wild type *F. pseudograminearum* CN23 was isolated from Jining City in China and maintained in our laboratory. The wild-type strain CN23 and all the transformants generated in this study were routinely cultured on potato dextrose agar (PDA) cultures at 25 °C for measuring growth rate and colony morphology, as previously described [26]. Conidiation and conidia length were assayed with 3-day-old liquid carboxymethylcellulose (CMC) cultures. The radial growth on PDA cultures supplemented with 0.02% SDS, 0.05% H_2_O_2_, 1.2 M NaCl, 300 μg mL^−1^ Congo red, 0.2 μg mL^−1^ tebuconazole, 0.08 μg mL^−1^ fludioxonil, 0.25 μg mL^−1^ phenamacril, and 0.25 μg mL^−1^ carbendazim was measured to assay sensitivity to various stresses and fungicides. Hyphae from the wild-type strain CN23 cultured in YEPD cultures for 12 h were collected. The cell walls of the hyphae were lysed using driselase and lysing enzymes to prepare protoplasts of the wild-type strain. Protoplasts were used for PEG-mediated transformation with 300 μg/mL hygromycin B (CalBiochem, La Jolla, CA, USA) for selection, as previously described [27]. Transformants were selected on hygromycin-resistant plates for subsequent verification.

### 2.3. Generation of the FpH3^K9R^, FpH3^K14R^, FpH3^K18R^, and FpH3^K23R^ Mutants

All FpH3 lysine-to-arginine mutation transformants were generated with the split-marker approach, as previously described [27]. Polymerase chain reaction (PCR) was performed with primer pairs HYG-F/HY-R and YG-F/HYG-R (Supplementary Table S1) to, respectively, amplify the upstream and downstream *hph* cassettes using the plasmid pcb1003 as a template. For FpH3K9 site, the 1.7 kb upstream flanking FpH3^K9R^ mutant allele was generated by overlapping PCR with primer pairs FpH3M-1F/FpH3K9R-R and FpH3K9R-F/FpH3M-2R (Supplementary Table S1), which were then fused with the hygromycin phosphotransferase (*hph*) cassette (Supplementary Figure S1). Primers FpH3K9R-R and FpH3K9R-F (Supplementary Table S1) contained the AA^29^G to AG^29^G mutation. Similarly, the 0.9 kb downstream flanking sequence was amplified with primer pairs FpH3M-3F/FpH3M-4R (Supplementary Table S1) and also fused with the *hph* cassette by overlapping PCR. The resulting upstream and downstream flanking sequences were transformed into protoplasts of CN23. Hygromycin-resistant transformants were screened using PCR and further confirmed through sequencing. The same approach was used to generate the FpH3^K14R^, FpH3^K18R^, and FpH3^K23R^ mutants.

### 2.4. Infection Assays with Wheat Seedling

An appropriate amount of wheat seeds (variety: Jimai 22) was taken and disinfected with 75% ethanol and 3% sodium hypochlorite for 30 s and 3 min, respectively. The seeds were rinsed three times with sterile water, and then the surface-disinfected wheat seeds were placed in a 25 °C incubator to germinate for 4 days. The stem bases of wheat seedlings were inoculated with 5 mm mycelium agar disks of the wild-type and mutant strains. These disks were securely attached to the wheat seedlings by rolling them with sterile gauze. Each piece of gauze contained 10 wheat seedlings, and each strain was repeated 3 times. Wheat seedlings inoculated with un-inoculated PDA disks served as the negative control. The wheat seedlings were placed in a plastic container and the container was sealed with cling film. Water was added to the container to achieve high humidity inside. The treated wheat seedlings were grown at 25 °C under a 12 h light/12 h dark photoperiod. Necrotic lesions on the stem bases were measured at 7 days post inoculation (dpi).

### 2.5. Assays for Wheat Head Infection and DON Production

Flowering wheat heads from the wheat cultivar Jimai 22 were cultivated at the Jiyang experimental demonstration base of Shandong Academy of Agricultural Sciences. The wild-type and all mutant strains were cultured in CMC broth on a shaker. The concentration of the conidium suspensions of all strains was adjusted to 1 × 10^5^ per milliliter. Flowering wheat heads were drop-inoculated with 10 μL of conidium suspensions of wild-type and mutant strains onto the third spikelet from the bottom, as previously described [28]. Water was sprayed onto the wheat spikes, and the inoculated wheat heads were covered with plastic bags for 48 h to maintain the humidity [29]. Symptoms of wheat head blight were examined 14 dpi, and the inoculated wheat kernels were harvested and analyzed for DON production, as previously described [30]. The inoculated wheat kernels were then placed in a drying oven at 50 °C for 48 h and weighed. Subsequently, they were transferred to centrifuge tubes, and acetonitrile/water (84/16) was added. The tubes were then shaken at 175 rpm on a shaker for 24 h. The extract was filtered through a 0.22-micron filter membrane, and the DON toxin content was determined using liquid chromatography–mass spectrometry/mass spectrometry (LC-MS/MS).

### 2.6. RNA-Seq Analysis

RNA samples were isolated from 24 h hyphae grown in YEPD (Yeast Extract Peptone Dextrose) cultures of wild-type and mutant strains using the TRIzol reagent (Invitrogen, Waltham, MA, USA). Total RNA was isolated from two biological replicates for each strain. RNA-seq libraries were generated using the NEB Next Ultra RNA Library Prep Kit (NEB, Ipswich, MA, USA) for Illumina following the manufacturer’s instructions. Briefly, mRNA was purified from total RNA using poly-T oligo-attached magnetic beads. After fragmentation, the first-strand cDNA was synthesized using random hexamer primers. Then, the second-strand cDNA was synthesized using dUTP, instead of dTTP. The directional library was ready after end repair, A-tailing, adapter ligation, size selection, USER enzyme digestion, amplification, and purification. Sequencing was performed on an Illumina platform using the paired-end 2 × 150 bp model at the Novogene Bioinformatics Institute (Beijing, China). For each sample, at least 24 Mb of paired-end reads were obtained. The resulting RNA-seq reads were aligned to the reference genome of *F. pseudograminearum* strain CS3096 [31]. Differential expression analysis was conducted using DEseq2 in R 4.3.0 [32] based on transcript abundance calculated by salmon [33]. Transcripts with Padj value below 0.05 and a |log_2_FC (fold change)| above 1 were regarded as differentially expressed genes. GO enrichment analysis was performed using software Blast2GO 5.2.5 [34].

## 3. Results

### 3.1. The Histone H3 N-Terminal Lysine Is Conserved from Fungi to Mammals

**Figure 1:**
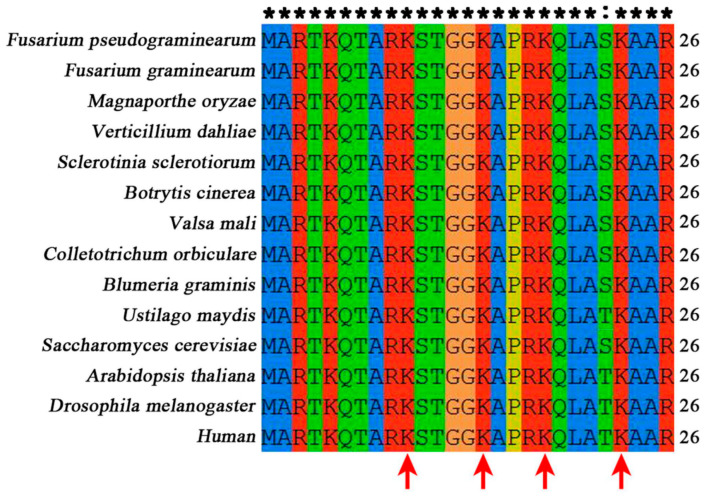
N-terminal sequence alignment of FpH3 with orthologs from representative species across fungi, plants, metazoans, and mammals. The alignment of N-terminal amino acid sequences of FpH3 and its orthologs from *Fusarium graminearum*, *Magnaporthe oryzae*, *Verticillium dahlia*, *Sclerotinia sclerotiorum*, *Botrytis cinerea*, *Valsa mali*, *Colletotrichum orbiculare*, *Blumeria graminis*, *Ustilago maydis*, *Puccinia graminis*, *Saccharomyces cerevisiae*, *Arabidopsis thaliana*, *Drosophila melanogaster*, and Human was performed using ClustalX 2.1 software. Conserved N-terminal lysines, mainly utilized for acetylation, are indicated by red arrows. Both the asterisk (*) and colon (:) represent conserved amino acids.

The predicted gene FPSE_04811 in *F. pseudograminearum* is the ortholog of *S. cerevisiae* histone H3 (HHT1 YBR010W and HHT2 YNL031C). In *S. cerevisiae*, two genes encode histone H3, whereas in *F. pseudograminearum*, only one gene encodes histone H3 (Supplementary Figure S2 and Supplementary Table S2). The amino acid identity between FpH3 and H3 orthologs in fungi, plants, metazoans, and mammals is all more than 88% (Supplementary Table S2). The amino acid sequence alignment analysis of FpH3 orthologs showed that histone acetylation sites K9, K14, K18, and K23 at the N-terminal of histone H3 are highly conserved in fungi, plants, metazoans, and mammals (Figure 1).

### 3.2. FpH3K9, K14, K18, and K23 Are Important for Vegetative Growth in F. pseudograminearum

To clarify the function of individual acetylation sites of histone H3 N-terminal in *F. pseudograminearum*, we generated FpH3^K9R^, FpH3^K14R^, FpH3^K18R^, and FpH3^K23R^ mutants using a homology recombination method (Supplementary Figure S1). The resulting FpH3^K9R^, FpH3^K14R^, FpH3^K18R^, and FpH3^K23R^ transformants with hygromycin resistance were confirmed by means of PCR assays and sequencing analysis, indicating the successful mutation of all target lysines while the wild type remained unchanged (Supplementary Figure S3).

**Figure 2:**
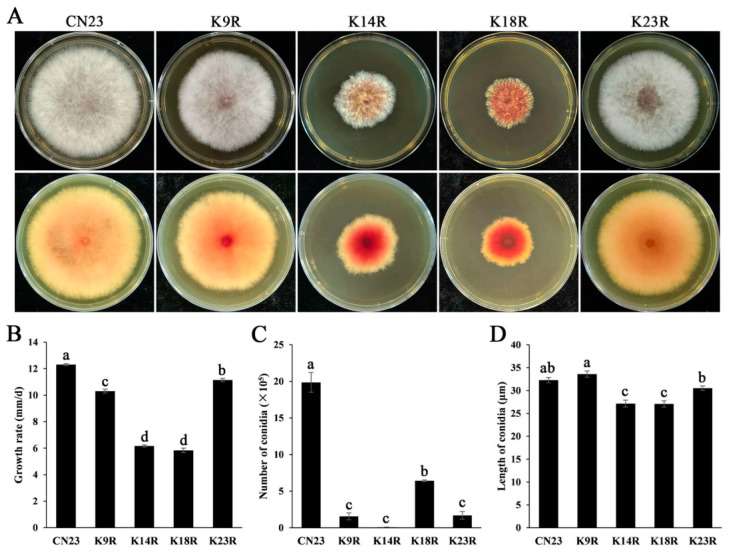
Defects in growth and conidiation in FpH3 N-terminal K to R mutants. (**A**) Three-day-old PDA cultures of the wild-type (CN23), FpH3^K9R^ (K9R), FpH3^K14R^ (K14R), FpH3^K18R^ (K18R), and FpH3^K23R^ (K23R) mutant strains. (**B**) Growth rate of CN23, K9R, K14R, K18R, and K23R strains. (**C**) Conidia numbers harvested from 3-day-old CMC cultures of CN23, K9R, K14R, K18R, and K23R strains. (**D**) Conidia length harvested from 3-day-old CMC cultures of CN23, K9R, K14R, K18R, and K23R strains. Different letters indicate significant differences based on ANOVA analysis with Duncan’s pair-wise comparison (*p* = 0.05).

When cultured on PDA cultures, the growth rate of FpH3^K9R^, FpH3^K14R^, FpH3^K18R^, and FpH3^K23R^ mutants decreased by 17.43%, 53.51%, 56.42%, and 10.17%, respectively, compared to the wild-type CN23 (Figure 2A, and Supplementary Table S3). Among them, the FpH3^K14R^ and FpH3^K18R^ mutants displayed severe defects and produced more pigment on PDA cultures, while the FpH3^K18R^ mutant produced fewer aerial hyphae compared to the wild type (Figure 2A). Therefore, mutations in FpH3K9, K14, K18, and K23 lead to significant defects during vegetative growth.

### 3.3. FpH3K9, K14, K18, and K23 Are Involved in Conidiation

Conidiation assays revealed that all the FpH3^K9R^, FpH3^K14R^, FpH3^K18R^, and FpH3^K23R^ mutants produced significantly fewer conidia than the wild type after shaking in liquid CMC cultures for 3 days (Figure 2B). Among them, the FpH3^K14R^ mutant produced few conidia (Figure 2B). Additionally, the length of conidia of the FpH3^K9R^, FpH3^K14R^, FpH3^K18R^, and FpH3^K23R^ mutants was measured. The FpH3^K9R^ and FpH3^K23R^ mutants’ conidia were about the same length as the wild type; however, the conidia length of the FpH3^K14R^ and FpH3^K18R^ mutants were significantly shorter than the wild type (Figure 2C). These results indicate that FpH3 N-terminal lysine sites are important for conidiation in *F. pseudograminearum*.

### 3.4. FpH3K14, K18, and K23 Are Required for Regulating Sensitivity to Different Stresses

**Figure 3:**
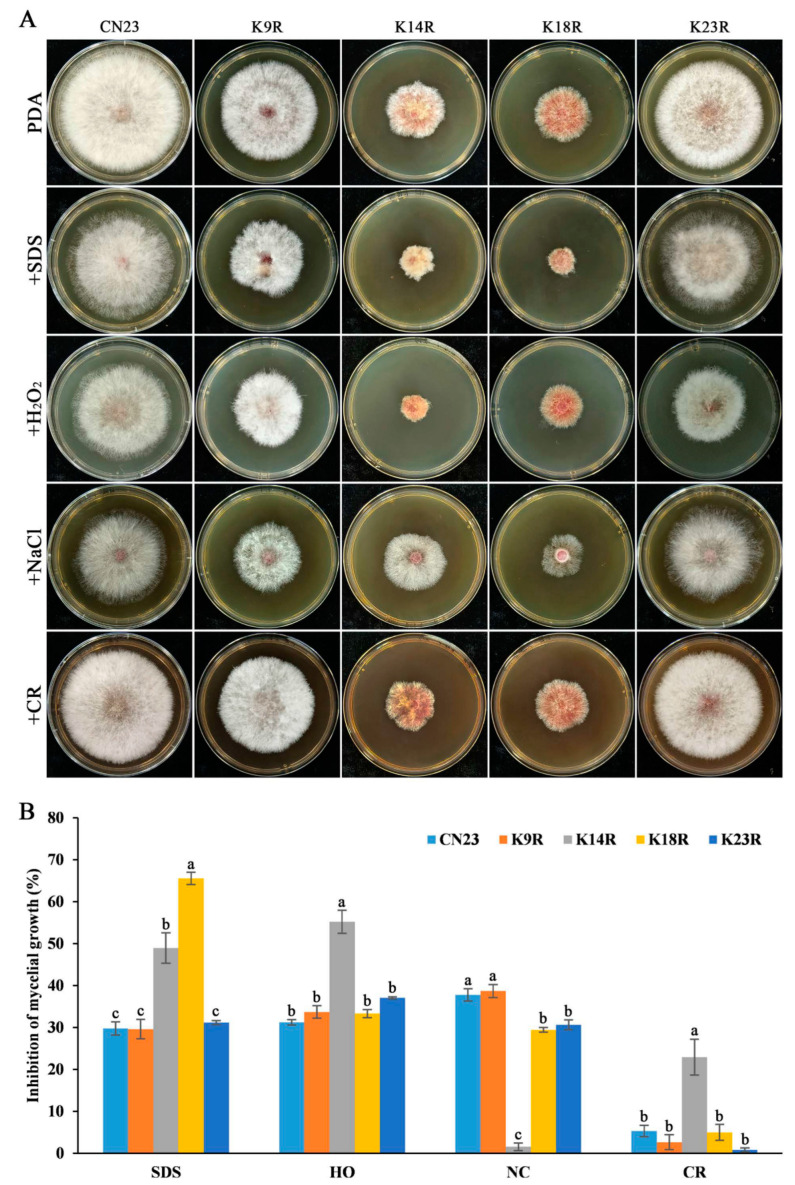
Morphology characteristics and mycelial inhibition of the FpH3 K to R mutants under various cellular stresses. (**A**) Three-day-old cultures of the wild-type (CN23), FpH3^K9R^ (K9R), FpH3^K14R^ (K14R), FpH3^K18R^ (K18R), and FpH3^K23R^ (K23R) mutant strains grown on regular PDA and PDA supplemented with 0.02% SDS, 0.05% H_2_O_2_ (HO), 1.2 M NaCl (NC), or 300 μg mv^−1^ Congo red (CR). (**B**) The percentage of mycelial growth inhibition of CN23, K9R, K14R, K18R, and K23R in PDA cultures with SDS, HO, NC, and CR compared to that in PDA cultures without stresses. Different letters denote significant differences based on ANOVA analysis with Duncan’s pair-wise comparison (*p* = 0.05).

To investigate the influence of FpH3 N-terminal lysine acetylation on stress sensitivity in *F. pseudograminearum*, we cultured the FpH3^K9R^, FpH3^K14R^, FpH3^K18R^, and FpH3^K23R^ mutants on PDA cultures with final concentrations of 0.02% SDS, 0.05% H_2_O_2_, 1.2M NaCl, and 300 μg mL^−1^ Congo red. The FpH3^K14R^ mutant displayed significantly increased sensitivity to SDS, H_2_O_2_, and Congo red and decreased sensitivity to NaCl (Figure 3A,B). Similarly, the FpH3^K18R^ mutant exhibited significantly increased sensitivity to SDS and decreased sensitivity to NaCl (Figure 3A,B). Additionally, the FpH3^K23R^ mutant showed significantly reduced sensitivity to NaCl and Congo red (Figure 3A,B). However, the FpH3^K9R^ mutant showed no significant changes in sensitivity to these stresses (Figure 3A,B). These findings suggest that FpH3K14, K18, and K23 are involved in response to reactive oxygen species (ROS) (SDS and H_2_O_2_), cell wall (Congo red), and osmotic (NaCl) stresses in *F. pseudograminearum*.

### 3.5. FpH3K9, K14, and K23 Regulate Sensitivity to Fungicides

**Figure 4:**
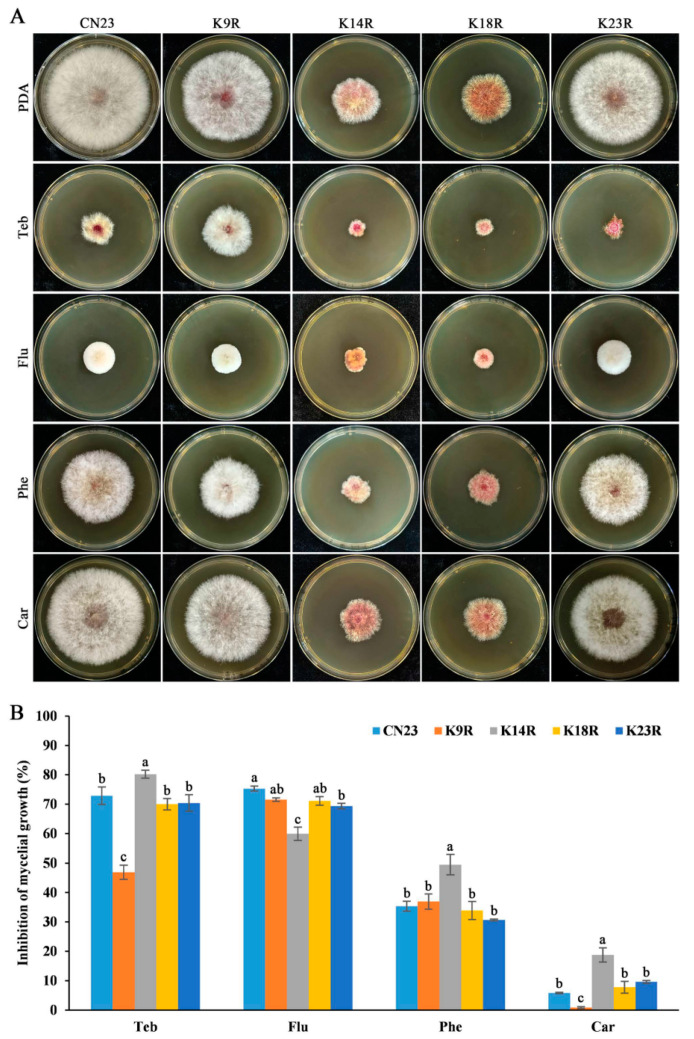
Sensitivity of FpH3 K to R mutants to different fungicides. (**A**) Three-day-old cultures of the wild-type (CN23), FpH3^K9R^ (K9R), FpH3^K14R^ (K14R), FpH3^K18R^ (K18R), and FpH3^K23R^ (K23R) mutant strains grown on regular PDA and PDA supplemented with 0.25 μg mL^−1^ tebuconazole (Teb), 0.08 μg mL^−1^ fludioxonil (Flu), 0.25 μg mL^−1^ phenamacril (Phe), or 0.25 μg mL^−1^ carbendazim (Car). (**B**) The percentage of mycelial growth inhibition of CN23, K9R, K14R, K18R, and K23R in PDA cultures with Teb, Flu, Phe, and Car compared to that in PDA cultures without fungicides. Different letters denote significant differences based on ANOVA analysis with Duncan’s pair-wise comparison (*p* = 0.05).

Seed coating with fungicides is currently the most cost-effective method for controlling FCR. To elucidate the function of lysine residues at the N-terminus of histone H3 in fungicide sensitivity in *F. pseudograminearum*, we cultured the FpH3^K9R^, FpH3^K14R^, FpH3^K18R^, and FpH3^K23R^ mutants in PDA cultures with final concentrations of 0.2 μg mL^−1^ tebuconazole, 0.08 μg mL^−1^ fludioxonile, 0.25 μg mL^−1^ phenamacril, and 0.25 μg mL^−1^ carbendazim. The FpH3^K9R^ mutant showed decreased sensitivity to tebuconazole and carbendazim (Figure 4A,B). Conversely, the FpH3^K14R^ mutant exhibited decreased sensitivity to fludioxonile but increased sensitivity to phenamacril and carbendazim (Figure 4A,B). The FpH3^K23R^ mutant displayed increased sensitivity only to carbendazim (Figure 4A,B). These results indicate that H3K9, K14, and K23 are required for the tolerance of *F. pseudograminearum* to tebuconazole, fludioxonile, phenamacril, and carbendazim.

### 3.6. FpH3K14 and FpH3K18 Are Crucial for Plant Infection

**Figure 5:**
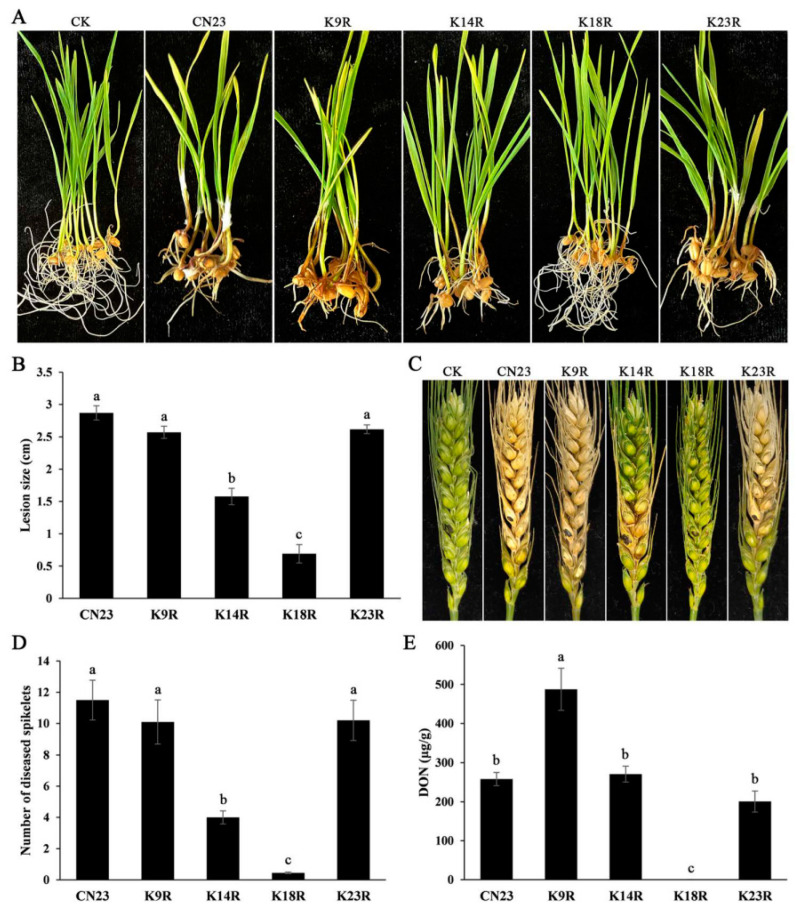
Defects of FpH3 K to R mutants in plant infection. (**A**) Wheat seedlings inoculated with the wild-type (CN23), FpH3^K9R^ (K9R), FpH3^K14R^ (K14R), FpH3^K18R^ (K18R), and FpH3^K23R^ (K23R) mutant strains were photographed at 7 dpi. CK were inoculated with blank PDA cultures. (**B**) The lesion size on wheat seedling stems caused by CN23, K9R, K14R, K18R, and K23R strains. (**C**) Flowering wheat heads inoculated with CN23, K9R, K14R, K18R, and K23R strains were photographed at 14 dpi. CK were inoculated with sterile water. (**D**) The number of diseased spikelets caused by CN23, K9R, K14R, K18R, and K23R strains. (**E**) DON levels in diseased wheat spikelets inoculated with CN23, K9R, K14R, K18R, and K23R strains. Different letters denote significant differences based on ANOVA analysis with Duncan’s pair-wise comparison (*p* = 0.05).

To clarify the role of K9, K14, K18, and K23 sites during infection in *F. pseudograminearum*, we conducted inoculation experiments on wheat seedling stem bases and heads. In infection assays with wheat seedlings, the FpH3^K14R^ and FpH3^K18R^ mutants caused significantly shorter brown lesions on wheat seedling stems compared to the wild type (Figure 5A,B). Similarly, in assays with flowering wheat heads, the wild type caused typical wheat scab symptoms with a diseased index of 11.5 (Figure 5C,D). The average diseased index of FpH3^K14R^ mutant was 4 (Figure 5D), which was significantly lower compared to the wild type. However, the FpH3^K18R^ mutant only caused symptoms on inoculated kernels and failed to spread to neighboring spikelets (Figure 5C). The FpH3^K9R^ and FpH3^K23R^ mutants showed no significant differences compared to the wild type in inoculation experiments at the wheat seedling stem bases and heads (Figure 5C,D). These findings suggest that FpH3K14 and K18 play critical roles in regulating plant infection.

### 3.7. FpH3K18R Mutant Is Defective in DON Production

DON is an important factor for the virulence of *F. pseudograminearum*. Since the FpH3^K14R^ and FpH3^K18R^ mutants displayed reduced pathogenicity, we assayed DON production in inoculated wheat kernels. The results showed that the FpH3^K18R^ mutant exhibited severe defects in DON biosynthesis (Figure 5E), whereas the FpH3^K14R^ mutant did not exhibit defects in DON production despite reduced virulence (Figure 5D,E). Interestingly, although the pathogenicity of the FpH3^K9R^ mutant remained unchanged, it showed a twofold increase in DON production compared to the wild type (Figure 5D,E). These results suggest that FpH3K9, K14, and K18 play various roles in the regulation of DON biosynthesis in *F. pseudograminearum*.

### 3.8. RNA-Seq Analysis with the FpH3K9R, FpH3K14R, FpH3K18R, and FpH3K23R Mutants

**Figure 6:**
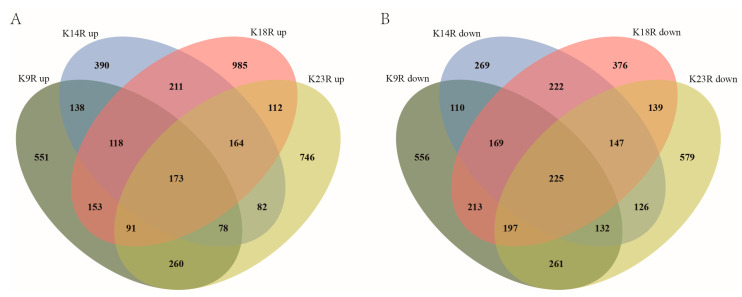
RNA-seq analysis of K9R, K14R, K18R, and K23R mutants. (**A**) Venn diagram illustrating the number of genes upregulated in the FpH3^K9R^ (K9R), FpH3^K14R^ (K14R), FpH3^K18R^ (K18R), and FpH3^K23R^ (K23R) mutant strains. (**B**) Venn diagram illustrating the number of genes downregulated in the K9R, K14R, K18R, and K23R strains.

**Figure 7:**
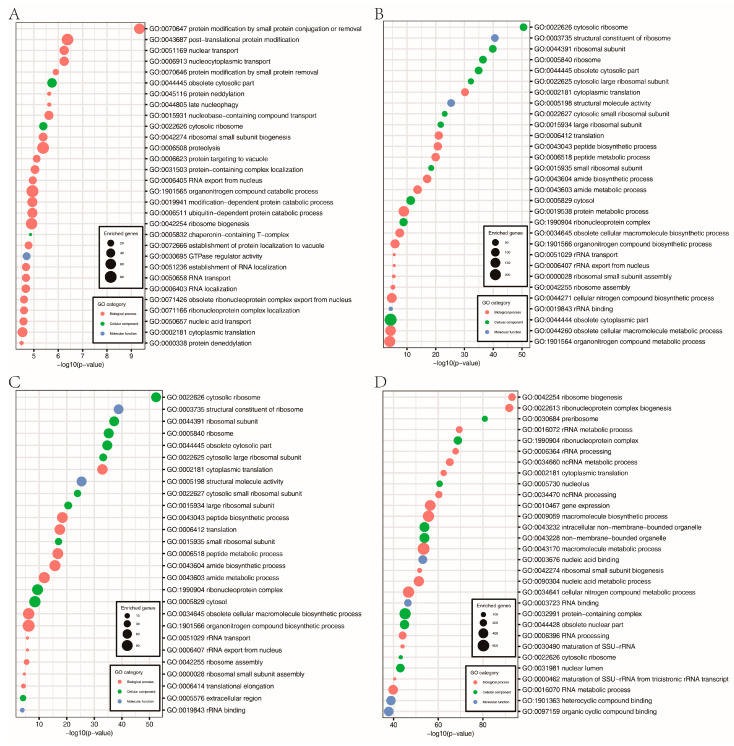
GO enrichment analysis of the upregulated genes in FpH3^K9R^ mutant (**A**), FpH3^K14R^ mutant (**B**), FpH3^K18R^ mutant (**C**), and FpH3^K23R^ mutant (**D**) strains.

Histone acetylation is closely associated with gene expression. Therefore, we performed RNA-seq analysis with RNA isolated from hyphae to identify genes affected by FpH3K9R, K14R, K18R, and K23R mutations during hyphal stage. In comparison with the wild type, 1562, 1354, 2007, and 1706 genes were upregulated 2-fold or more in the FpH3^K9R^, FpH3^K14R^, FpH3^K18R^, and FpH3^K23R^ mutants, respectively (Figure 6A and Supplementary Tables S4–S7). GO enrichment analysis revealed distinct functional enrichments among the upregulated genes. Specifically, genes upregulated in the FpH3^K9R^ mutant were associated with post-translational protein modification, protein neddylation, proteolysis, protein targeting to vacuole, and modification-dependent protein catabolic process (Figure 7A). Similarly, genes upregulated in the FpH3^K14R^ and FpH3^K18R^ mutants were enriched for functions related to cytosolic ribosome, cytoplasmic translation, the biosynthetic and metabolic process of peptide and amide, and rRNA export from nucleus (Figure 7B,C). Genes upregulated in the FpH3^K23R^ mutant were associated with ribonucleoprotein complex biogenesis, rRNA processing, cytoplasmic translation, gene expression, and macromolecule biosynthetic process (Figure 7D).

**Figure 8:**
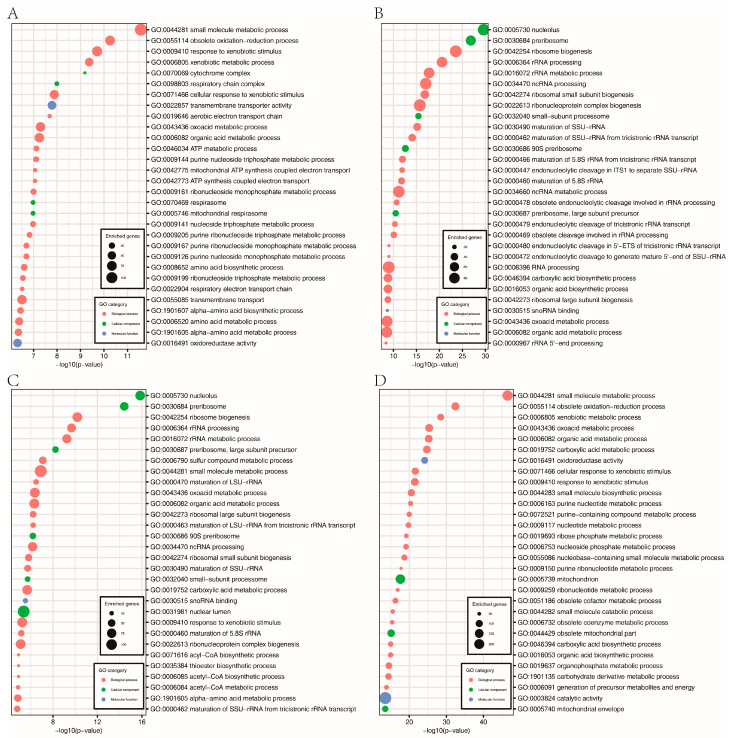
GO enrichment analysis of the downregulated genes in the FpH3^K9R^ mutant (**A**), FpH3^K14R^ mutant (**B**), FpH3^K18R^ mutant (**C**), and FpH3^K23R^ mutant (**D**) strains.

Furthermore, a total of 1863, 1400, 1688, and 1806 genes were downregulated in the FpH3^K9R^, FpH3^K14R^, FpH3^K18R^, and FpH3^K23R^ mutants, respectively, compared to the wild type (Figure 6B and Supplementary Tables S4–S7). GO enrichment analysis revealed specific functional enrichments among the downregulated genes. Genes downregulated in the FpH3^K9R^ mutant were associated with processes such as cytochrome complex assembly, aerobic electron transport chain, and ATP metabolic processes (Figure 8A). Similarly, genes downregulated in the FpH3^K14R^ and FpH3^K18R^ mutants showed enrichment in functions related to ribosome biogenesis, rRNA processing, ribonucleoprotein complex biogenesis, and the maturation of SSU-rRNA and 5.8S rRNA (Figure 8B,C). Genes downregulated in the FpH3^K23R^ mutant were enriched for various metabolic processes involving small molecule, organic acid, purine nucleotide, and ribose phosphate (Figure 8D). Histone H3 K9, K14, K18, and K23 may influence cell growth and development by regulating the expression of genes associated with relevant biological processes, molecular functions, and cellular components (Supplementary Figure S4).

Numerous genes crucial for growth and pathogenicity were found to be downregulated in the FpH3^K9R^, FpH3^K14R^, FpH3^K18R^, and FpH3^K23R^ mutants. For instance, peroxin (FPSE_11808, *FpPEX4*) and beta tubulin (FPSE_03337, *FpTUB1*) (Supplementary Table S4), which are important for growth and conidiation, were downregulated in the FpH3^K9R^ mutant [35,36,37]. Additionally, the essential protein kinase (FPSE_01027, *FpMPS1*) (Supplementary Table S4) was also downregulated in the FpH3^K9R^ mutant [38]. In the FpH3^K14R^ mutant, downregulated genes such as histone acetyltransferase (FPSE_05132, *FpELP3*), kinase (FPSE_04786, *FpSAT4*), and arginine methyltransferase (FPSE_01029, *FpAMT1*) (Supplementary Table S5) are involved in regulating growth, pathogenicity, and DON biosynthesis [38,39,40]. Similarly, the cytochrome P450 monooxygenase (FPSE_10099) and protein kinase (FPSE_01608, *FpKNS1*), downregulated in the FpH3^K14R^ mutant (Supplementary Table S5), are essential genes [38,41]. Furthermore, genes downregulated in the FpH3^K18R^ mutant, including ABC-C transporter (FPSE_04130, *FpABCC9*), AreA transcription factor (FPSE_10693, *FpAREA*), and phosphorylated transcription factor (FPSE_02607, *FpSR*), (Supplementary Table S6), are crucial for growth, pathogenicity, and DON production [42,43,44]. Likewise, genes downregulated in the FpH3^K18R^ mutant, such as TOR signaling pathway regulator (FPSE_00842, *FpTAP42*) and two protein kinases (FPSE_04764 (*FpIPL1*) and FPSE_05456 (*FpTOR*)) (Supplementary Table S6) are essential genes [38,45]. Additionally, the FpH3^K23R^ mutant downregulated gene, including threonine deaminase (FPSE_08771, *FpILV1*) and acetohydroxyacid synthase (FPSE_09041, *FpILV2*) (Supplementary Table S7), are crucial for growth and conidiation [46,47].

Another subset of important genes is simultaneously downregulated in multiple mutants. Transcription factor (FPSE_05044, *FpRLM1*), phosphatidate phosphatase (FPSE_11643, *FpPAH1*), and Nem1-Spo7 phosphatase complex regulatory subunit (FPSE_04899, *FpSPO7*), downregulated in both the FpH3^K14R^ and FpH3^K18R^ mutants (Supplementary Tables S5 and S6), are crucial for growth, pathogenicity, and DON production [48,49]. Similarly, the NuA4 acetyltransferase complex catalytic subunit (FPSE_08733, *FpESA1*) and protein kinase (FPSE_01755, *FpRIO1*), essential genes, are also downregulated in both the FpH3^K14R^ and FpH3^K18R^ mutants (Supplementary Tables S5 and S6) [50]. Furthermore, phosphatase (FPSE_01238, *FpCDC14*), which is downregulated in the FpH3^K9R^, FpH3^K14R^, and FpH3^K18R^ mutants (Supplementary Tables S4–S6), regulates growth, conidiation, pathogenicity, and DON production [51]. The essential kinase (FPSE_07936, *FpNIMA*) is also downregulated in the FpH3^K9R^, FpH3^K14R^, and FpH3^K18R^ mutants (Supplementary Tables S4–S6) [38]. Additionally, dihydroxyacid dehydratase (FPSE_05147, *FpILV3A*), hexokinase (FPSE_04405, *FpHXK1*), and acetohydroxyacid synthase (FPSE_00996, *FpILV6*), which play vital roles in growth, conidiation, and pathogenicity, are all downregulated in the FpH3^K9R^, FpH3^K14R^, and FpH3^K23R^ mutants (Supplementary Tables S4, S5 and S7) [47,52,53]. The reduction in the expression of these genes may partly account for the defects observed in the FpH3^K9R^, FpH3^K14R^, FpH3^K18R^, and FpH3^K23R^ mutants.

## 4. Discussion

Unlike *S. cerevisiae*, which possesses two copies of histone H3 gene, the histone H3 gene exists as a single copy in *F. pseudograminearum*. Altering any of the conserved lysine residues in FpH3 results in severe functional defects. The loss of certain HATs always leads to reduced histone H3 acetylation levels, thereby influencing hyphal growth, plant infection, and gene expression. For instance, in *F. graminearum*, the acetylation levels of H3K9, K14, and K18 were decreased in the *Fggcn5* mutant, with a similar decrease observed in H3K14 acetylation in the *Fgsas3* mutant [15]. In this study, both FpH3^K14R^ and FpH3^K18R^ mutants exhibited severe defects in growth, conidiation, and virulence in *F. pseudograminearum*. Notably, the defects observed in the *Fggcn5* mutant were more pronounced compared to the *Fgsas3* mutant in growth, conidiation, sexual reproduction, and pathogenicity [15]. This discrepancy might be attributed to the simultaneous regulation of H3K9, K14, and K18 acetylation by the *Fggcn5* mutant, whereas the *Fgsas3* mutant primarily influences H3K14 acetylation. Interestingly, the *Ffgcn5* mutant of *F. fujikuroi* displayed low H3K9 and K18 acetylation levels but maintained a normal H3K14 acetylation level, indicating species-specific variations in acetylation patterns [15,24]. In *Z. tritici*, ZtSas3 is involved in the H3K9 and K14 acetylation during effector gene activation in plant infection [17], suggesting a potential role in the reduced pathogenicity observed in the FpH3^K14R^ mutant in *F. pseudograminearum*.

The *Fggcn5* and *Fgsas3* mutants both exhibited significantly increased sensitivity to H_2_O_2_ in *F. graminearum* [15]. Similarly, in *Z. tritici*, the *Ztsas3* mutant displayed increased sensitivity to H_2_O_2_ and Congo red [17]. Additionally, in *Aspergillus flavus*, the *Aflrtt109* mutant also showed increased sensitivity to H_2_O_2_ and Congo red [54]. Likewise, the *Mogcn5* mutant demonstrated significantly increased sensitivity to H_2_O_2_ in *M. oryzae* [55]. Conversely, in *A. fumigatus*, the *Afelp3* mutant exhibited decreased sensitivity to Congo red [18]. In this study, we observed that the FpH3^K14R^ mutant displayed heightened sensitivity to both H_2_O_2_ and Congo red, consistent with findings in the *sas3* and *gcn5* mutants. This suggests that *GCN5*, *SAS3*, *RTT109*, and *ELP3* may play roles in H3K14 acetylation, thereby influencing sensitivity to H_2_O_2_ and Congo red. Furthermore, we found that the expression of *FpELP3* was downregulated in the FpH3^K14R^ mutant in *F. pseudograminearum*. This suggests that H3K14 may regulate sensitivity to Congo red by modulating the expression level of *ELP3*.

The homologous genes FPSE_00109, FPSE_09646, and FPSE_04130, corresponding to *FgCYP51A*, *FgNPC1*, and *FgABCC9* in *F. graminearum*, were all found to be upregulated in the FpH3^K9R^ mutant. The deletion of *FgCYP51A*, *FgNPC1*, and *FgABCC9* genes in *F. graminearum* resulted in increased sensitivity to tebuconazole [44,56,57]. In this study, we found the FpH3^K9R^ mutant showed decreased sensitivity to tebuconazole. This might be due to the upregulation of genes *FgCYP51A*, *FgNPC1*, and *FgABCC9*, leading to the decreased sensitivity of the FpH3^K9R^ mutant to tebuconazole. Additionally, FPSE_12291, the ortholog of *FgERG3B*, was downregulated in the FpH3^K14R^ mutant, while FPSE_01847, the ortholog of the gene *FgERG5B*, was upregulated in the same mutant. The deletion mutants of *FgERG3B* and *FgERG5B* in *F. graminearum* displayed increased sensitivity and decreased sensitivity to tebuconazole, respectively [58]. This likely explains the heightened sensitivity of the FpH3^K14R^ mutant to tebuconazole. Furthermore, FPSE_11531, the ortholog of VelB, was upregulated in the FpH3^K23R^ mutant. The *FgvelB* mutant exhibited increase sensitivity to fludioxonil [59]. Therefore, the upregulation of gene FPSE_11531 might contribute to the decreased sensitivity of the FpH3^K23R^ mutant to fludioxonil.

Histone acetylation is closely associated with gene transcription. In the FpH3^K9R^, FpH3^K14R^, FpH3^K18R^, and FpH3^K23R^ mutants, several genes crucial for growth and pathogenicity, as well as some essential genes, are downregulated. Among them, 10 protein kinase genes (*FpMPS1*, *FpKNS1*, *FpSAT4*, *FpIPL1*, *FpRIO1*, *FpCOT1*, *FpPRR2*, *FpNIMA*, *FpMGV1*, and *FpTOR*) are downregulated, with 6 of them being essential genes [38]. Additionally, four cytochrome P450 monooxygenase genes (FPSE_10099, FPSE_00539, FPSE_02139, and FPSE_09613) are downregulated [41]. Furthermore, five downregulated genes (*FpTAP42*, *FgNEM1*, *FgPAH1*, *FgSPO7*, and *FgACC1*) are involved in the rapamycin pathway which regulate growth, pathogenicity, and DON biosythesis [45,48]. Additionally, two acetyltransferases (*FpESA1* and *FpELP3*) and three crucial transcription factors (*FpAREA*, *FpSR*, *FpRLM1*) are downregulated [40,42,43,49]. Given the significant role of histone H3 N-terminal acetylation in regulating the expression of many crucial genes, it holds considerable importance in cellular processes.

## 5. Conclusions

In this study, we functionally characterized four conserved lysine residues responsible for acetylation in the N-terminal region of FpH3. We discovered that mutations (K-to-R) occurring at these residues play vital roles in fungal development, pathogenicity, and gene expression in *F. pseudograminearum*. Furthermore, thousands of genes were found to be downregulated in the FpH3^K9R^, FpH3^K14R^, FpH3^K18R^, and FpH3^K23R^ mutants. FpH3K9 primarily regulated genes related to ATP metabolism, while FpH3K14 and FpH3K18 primarily regulated genes associated with ribosome biogenesis. Given the high conservation of the N-terminal lysine residues in H3, our findings may contribute to understanding the regulatory mechanism of H3 N-terminal acetylation in fungi, plants, metazoans, and mammals. Additionally, our results can provide a theoretical basis for the development of related fungicides and the study of human drug targets.

## Data Availability

The RNA-seq data generated in this study have been deposited in the NCBI Sequence Read Archive database under the accession code PRJNA657935.

## Supplementary Materials

The following supporting information can be downloaded at: https://www.mdpi.com/article/10.3390/jof10060379/s1, Figure S1: Schematic representation of primer design for generating FpH3 lysine-to-arginine mutation constructs; Figure S2: Phylogenetic analysis of histone H3 across fungi, plants, metazoans, and mammals; Figure S3: Sequencing analysis was performed to identify the mutation of A29G, A44G, A56G, and A71G in FpH3^K9R^, FpH3^K14R^, FpH3^K18R^, and FpH3^K23R^ mutants, respectively, compared to the wild type; Figure S4: Schematic diagram of histone H3 K9, K14, K18, and K23 regulation of cell growth and development; Table S1: Primers used in this study; Table S2: Amino acid identity analysis of histone H3 between *F. pseudograminearum* and other representative species; Table S3: The wild type and transformants of *Fusarium pseudograminearum* used in this study; Table S4: Identification of differentially expressed genes (DEGs) in the FpH3^K9R^ mutant; Table S5: Identification of differentially expressed genes (DEGs) in the FpH3^K14R^ mutant; Table S6: Identification of differentially expressed genes (DEGs) in the FpH3^K18R^ mutant; Table S7: Identification of differentially expressed genes (DEGs) in the FpH3^K23R^ mutant.
