# Supplementary material for: Histone H3 N-Terminal Lysine Acetylation Governs Fungal Growth, Conidiation, and Pathogenicity through Regulating Gene Expression in Fusarium pseudograminearum
Source: J Fungi (Basel). 2024 May 25;10(6):379. doi: 10.3390/jof10060379 (PMC11204548; doi:10.3390/jof10060379)
Supplement: Supplementary file 1 [file jof-10-00379-s001.zip › Table S1.pdf]

Table S1. Primers used in this study.

Primer names	Sequences (5’-3’)
FpH3M-1F	TGTTTGTGTGCTGGTGTGTTGAC
FpH3M-2R	TTGACCTCCACTAGCTCCAGCCAAGCCTATTCGCGAGCAAGACGCGTGTA
FpH3M-3F	GAATAGAGTAGATGCCGACCGCGGGTTGCACTTTCGCAAATGCCTTAACAG
FpH3M-4R	TTCCCAAGGTCCTTATGCTCAAC
FpH3M-5F	GGGATATCATGGTTAGGGGATAA
FpH3M-6R	ACTGTGTGACTTGCAACGTTCGT
FpH3M-7F	TGCACTGCTCATTATCTGCTT
FpH3M-8R	ATGATGCCGTACAGGAGGAGAT
FpH3K9R-F	GAAGGTCCACTGGTGGCAAGGCCCCTCGCAAGCAGCT
FpH3K9R-R	AGCTGCTTGCGAGGGGCCTTGCCACCAGTGGACCTTC
FpH3K14R-F	GAAAGTCCACTGGTGGCAGGGCCCCTCGCAAGCAGCT
FpH3K14R-R	AGCTGCTTGCGAGGGGCCCTGCCACCAGTGGACTTTC
FpH3K18R-F	GAAAGTCCACTGGTGGCAAGGCCCCTCGCAGGCAGCT
FpH3K18R-R	AGCTGCCTGCGAGGGGCCTTGCCACCAGTGGACTTTC
FpH3K23R-F	CTCGCAAGCAGCTCGCTTCCAGGGCTGGTAAGTTCTC
FpH3K23R-R	GAGAACTTACCAGCCCTGGAAGCGAGCTGCTTGCGAG
HYG-F	GGCTTGGCTGGAGCTAGTGGAGGTCAA
HY-R	GTATTGACCGATTCCTTGCGGTCCGAA
HYG-R	AACCCGCGGTCGGCATCTACTCTATTC
YG-F	GATGTAGGAGGGCGTGGATATGTCCT
H855-R	GCTGATCTGACCAGTTGC
H856-F	GTCGATGCGACGCAATCGT
H850	TTCCTCCCTTTATTTCAGATTCAA
H852	ATGTTGGCGACCTCGTATTGG
