# Supplementary material for: Histone H3 N-Terminal Lysine Acetylation Governs Fungal Growth, Conidiation, and Pathogenicity through Regulating Gene Expression in Fusarium pseudograminearum
Source: J Fungi (Basel). 2024 May 25;10(6):379. doi: 10.3390/jof10060379 (PMC11204548; doi:10.3390/jof10060379)
Supplement: Supplementary file 1 [file jof-10-00379-s001.zip › Table S2.pdf]

Table S2. Amino acid identity analysis of histone H3 between *F. pseudograminearum* and other representative species.

Species	Gene ID	Protein ID	Number of amino acids	Amino acid identity to FpH3 (%)
Fusarium graminearum	FGSG_04290	FgH3	136	100.00%
Magnaporthe oryzae	MGG_01159	MoH3	136	100.00%
Botrytis cinerea	BcDW1_8978	BcH3	136	98.53%
Sclerotinia sclerotiorum	SS1G_09608	SsH3	136	98.53%
Verticillium dahliae	VDAG_10035	VdH3	136	100.00%
Valsa mali	VM1G_09220	VmH3	136	100.00%
Colletotrichum orbiculare	Cob_04734	CoH3	136	100.00%
Ustilago maydis	UMAG_03916	UmH3.1	136	91.91%
Ustilago maydis	UMAG_02709	UmH3.2	136	91.18%
Blumeria graminis	BLGH_06097	BgH3.1	136	98.53%
Blumeria graminis	BLGH_06808	BgH3.2	136	98.53%
Saccharomyces cerevisiae	YBR010W	HHT1	136	94.85%
Saccharomyces cerevisiae	YNL031C	HHT2	136	94.85%
Arabidopsis thaliana	AT5G65360	AtH3.1	136	88.89%
Drosophila melanogaster	FBgn0014857	DmH3.3A	136	91.85%
Human	ENSG00000163041	H3-3A	136	91.85%
