# Supplementary material for: Histone H3 N-Terminal Lysine Acetylation Governs Fungal Growth, Conidiation, and Pathogenicity through Regulating Gene Expression in Fusarium pseudograminearum
Source: J Fungi (Basel). 2024 May 25;10(6):379. doi: 10.3390/jof10060379 (PMC11204548; doi:10.3390/jof10060379)
Supplement: Supplementary file 1 [file jof-10-00379-s001.zip › Table S3.pdf]

Table S3. The wild type and transformants of *Fusarium pseudograminearum* used in this study.

Strain	Brief description	Reference
CN23	Wild type	This study
K9R	$FgH3^{K9R}$ mutant of CN23	This study
K14R	$FgH3^{K14R}$ mutant of CN23	This study
K18R	$FgH3^{K18R}$ mutant of CN23	This study
K23R	$FgH3^{K23R}$ mutant of CN23	This study
