# Supplementary figures and images for: Histone H3 N-Terminal Lysine Acetylation Governs Fungal Growth, Conidiation, and Pathogenicity through Regulating Gene Expression in Fusarium pseudograminearum
Source: J Fungi (Basel). 2024 May 25;10(6):379. doi: 10.3390/jof10060379 (PMC11204548; doi:10.3390/jof10060379)

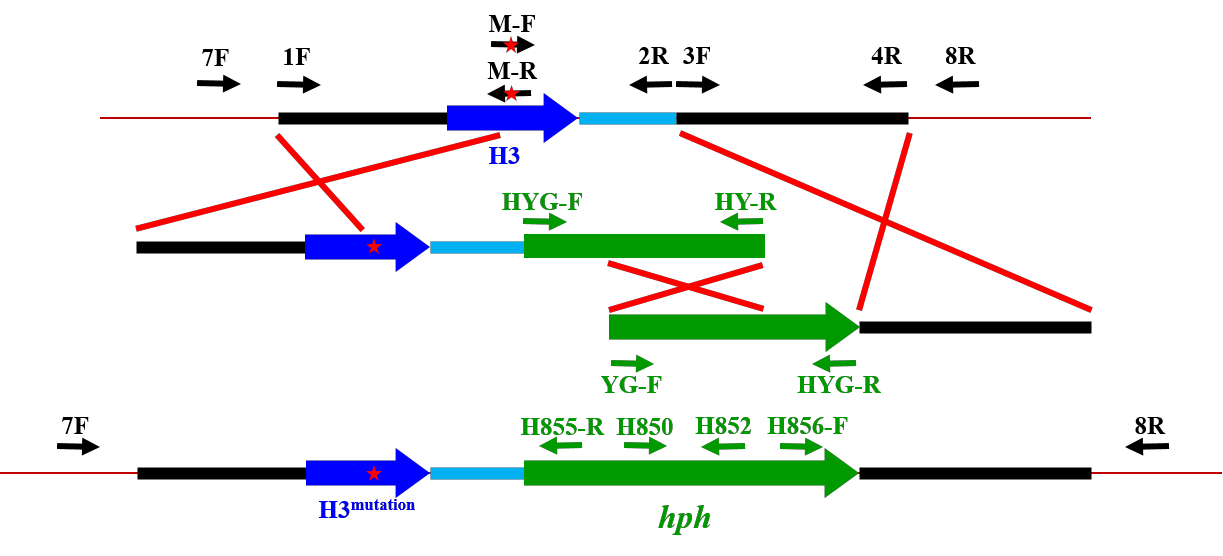

Supplement: Supplementary file 1 [file jof-10-00379-s001.zip › FigS1-lzw.tif]

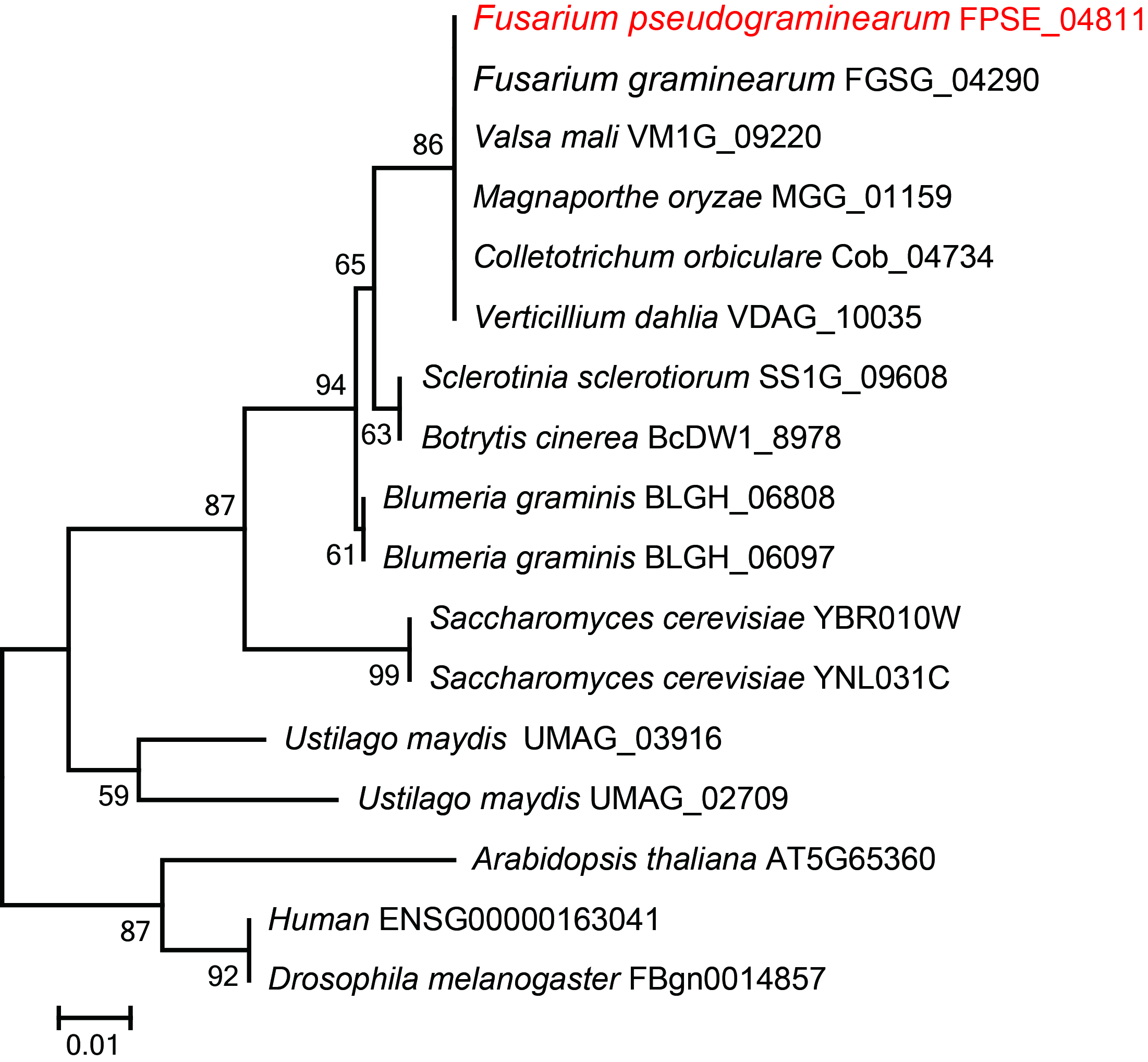

Supplement: Supplementary file 1 [file jof-10-00379-s001.zip › FigS2-lzw.tif]

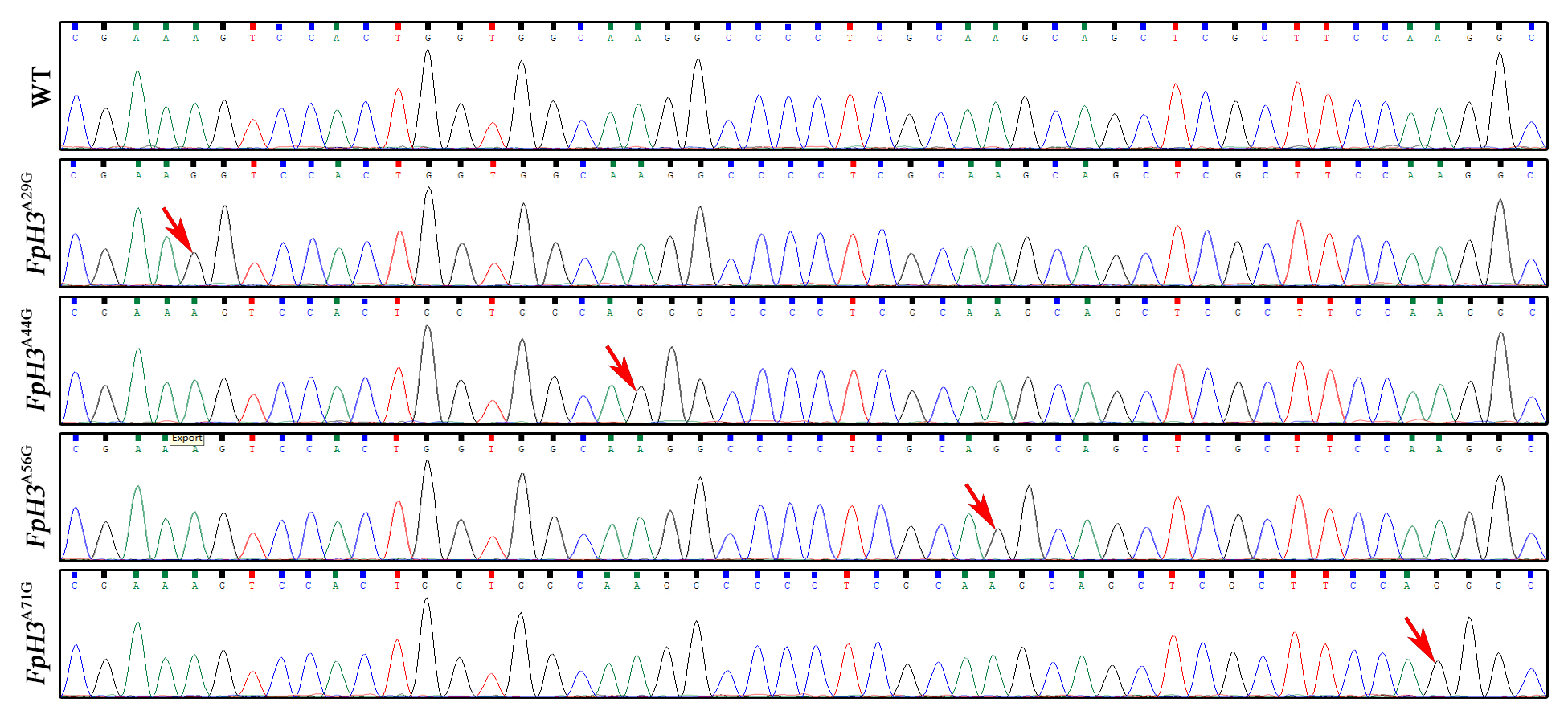

Supplement: Supplementary file 1 [file jof-10-00379-s001.zip › FigS3-lzw.tif]

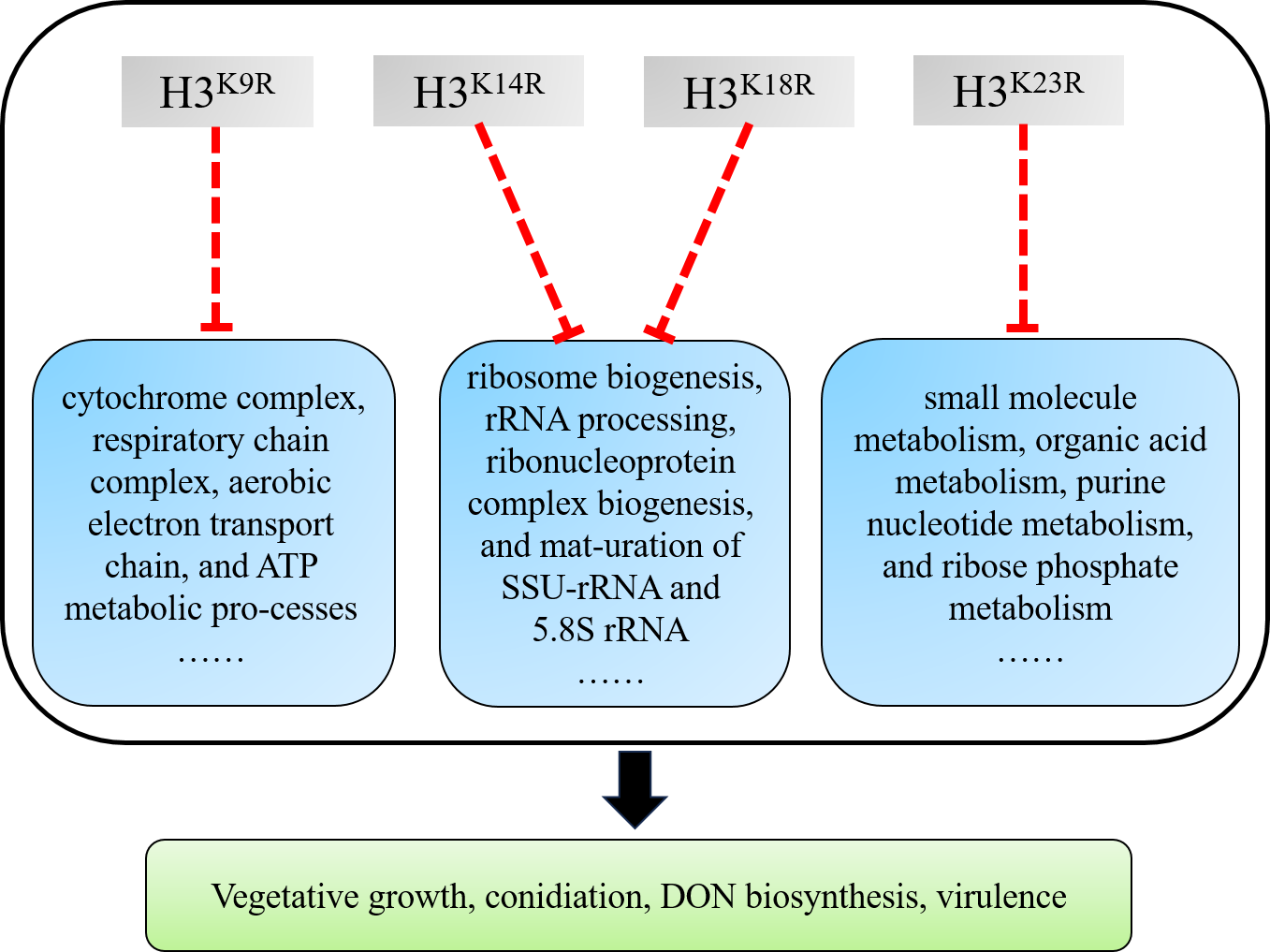

Supplement: Supplementary file 1 [file jof-10-00379-s001.zip › Figure S4.tif]
